# Association of premature ejaculation with adult attention deficit hyperactivity disorder: Relationship with digit ratio

**DOI:** 10.1097/MD.0000000000043167

**Published:** 2025-07-04

**Authors:** Gonca Ayse Unal, Selin Balki Tekin, Ayse Nur İnci Kenar, Sezai Ustun Aydin, Alper Ziblak

**Affiliations:** a Psychiatry Clinic, Mersin City Hospital, Mersin, Turkiye; b Psychiatry Clinic, Denizli State Hospital, Denizli, Turkiye; c Department of Psychiatry, School of Medicine, Pamukkale University, Denizli, Turkiye; d Department of Psychiatry, Rutgers New Jersey Medical School, Newark, NJ.

**Keywords:** attention deficit hyperactivity disorder, digit ratio, premature ejaculation

## Abstract

Increased intrauterine testosterone exposure may be a common etiological factor for attention deficit hyperactivity disorder (ADHD) and premature ejaculation (PE). This study aimed to determine the frequency of PE in adult ADHD and its relationship with the ratio between the second digit (2D) and fourth digit (4D) (2D:4D), which is accepted as an indicator of fetal testosterone exposure. 24 ADHD patients and 38 healthy controls were included in the study. Patients were evaluated using the Wender Utah Rating Scale (WURS) and Adult ADD (Attention Deficit Disorder)/ADHD DSM (The Diagnostic and Statistical Manual of Mental Disorders) IV-Based Diagnostic Screening and Rating Scale. PE was diagnosed according to DSM-5 criteria during the clinical interview with the participants. The 2D and 4D of the participants were measured directly with a digital caliper (0.01 mm sensitivity). The 2D:4D of the 2 groups was compared with each other. The prevalence of PE was 45.8% in the ADHD group and 5.2% in the control group. There was a statistically significant difference between the ADHD and control groups in terms of the 2D:4D (*P* = .004). Although there was no statistically significant difference (*P* > .05), ADHD patients with PE had lower right and left 2D:4D compared to those without PE. In adults with ADHD, PE was found to be both more frequent and lower in the 2D:4D, suggesting that high testosterone levels play a role in both the etiology of ADHD and PE. For this reason, it was thought that the frequency of coexistence is high because of the possible common etiology of these 2 clinical diagnoses.

## 1. Introduction

Attention deficit hyperactivity disorder (ADHD) is a neuropsychiatric disorder that begins in early childhood.^[1]^ The prevalence of ADHD is 5.3% in childhood and 1% to 4% in adulthood.^[2]^ Among the patients who were diagnosed with ADHD in childhood, it is reported that ADHD symptoms continue in 50% to 80% of them in adolescence and 30% to 50% of them in adulthood.^[3]^

Premature ejaculation (PE) is defined as an ejaculation that occurs before or immediately after vaginal penetration, which cannot be delayed and causes stress and avoidance of sexual intercourse.^[4]^ PE is the most common ejaculation disorder, with an incidence of 20% to 30%.^[5]^ To our knowledge, the relationship between adult ADHD and PE has been investigated in only 2 studies. In these studies, they reported that the percentage of those diagnosed with ADHD was significantly higher (42.1%, 45.83%) in patients with lifelong PE compared to the control groups (3.7%, 5.0%, respectively).^[6,7]^ However, both studies examined the frequency of ADHD in PE patients.

The psychopathology of PE is not fully understood and theories on this subject do not provide an adequate explanation. In addition to genetic and neurobiological features, psychological factors are also thought to be effective in the development of PE. It has been suggested that dopamine, oxytocin and serotonergic neurotransmitter systems may play a role in the etiology of both PE and ADHD. It has also been reported that dopamine receptor genes play a role in the etiology of both diseases.^[7]^

Testosterone is a hormone that controls ejaculation. Testosterone has a facilitating role in the control of the ejaculation reflex, regardless of central or peripheral problems, by shortening the latency of erection. It has been reported that patients with PE have higher free testosterone and follicle-stimulating hormone levels compared to normal men, which supports the facilitating effect of testosterone in the control of the ejaculation reflex.^[8]^ Some recent animal studies support the idea that prenatal testosterone exposure may play a role in the development of ADHD. It has been shown that male fetuses are exposed to significantly higher testosterone levels than female fetuses.^[9]^

However, recent evidence has shown that the 2D:4D is constant throughout life and is inversely proportional to fetal testosterone exposure.^[10]^ A negative relationship between the 2D:4D and the ratio of fetal testosterone to fetal estradiol is related to the effect of prenatal sex steroids on finger development. Low 2D:4D is associated with high fetal testosterone. The relationship reflects testosterone produced by the fetal gonads and adrenals, as the fetus is isolated from maternal androgen by conversion to estradiol within the placenta by the enzyme aromatase.^[10]^ Therefore, the 2D:4D was used as a noninvasive marker of fetal testosterone exposure in studies to investigate the relationship between prenatal testosterone exposure and neurodevelopmental disorders such as ADHD. Lower 2D:4D was associated with more externalizing problems and more ADHD symptoms, such as inattentiveness and impulsivity.^[11,12]^

Prenatal testosterone exposure and/or high testosterone levels might play a role in the etiology of both adult ADHD and PE. In addition, in a study conducted with patients who applied to an infertility clinic, a high testosterone level was found to be associated with a low 2D:4D.^[13]^

In light of this information, it is suggested that increased intrauterine testosterone exposure may be a common etiological factor for ADHD and PE. It was hypothesized that PE may be more frequent in individuals with ADHD and that the 2D:4D may be lower in these individuals. However, a study researching the relationship between ADHD and PE combined with the 2D:4D cannot be found as reported in the English Literature. This study aimed to determine the frequency of PE in adult ADHD patients and its relationship with the 2D:4D, which is accepted as an indicator of fetal testosterone exposure.

## 2. Materials and methods

### 2.1. Study design

This study was conducted following the principles of the Helsinki Declaration and approved by the Pamukkale University Noninterventional Clinical Research Ethics Committee (approval no:04, date: 23.02.2016). As a result of the power analysis, it was calculated that 90% power would be obtained with 95% confidence when there are at least 21 patients. Forty-three male patients diagnosed with ADHD who applied to the psychiatry outpatient clinic between March 2016 and June 2016 and were sexually active with a partner were included in the study. For the control group, 38 healthy males, similar in age to the patient group, were carefully selected among the relatives of the patients hospitalized in other clinics of our hospital. Exclusion criteria were having a neurological or chronic disease, a diagnosis of PE due to an organic etiology, mental retardation, or a psychiatric disorder in the context of a general medical condition such as epilepsy, multiple sclerosis, brain tumors etc. Patients who were diagnosed with adult ADHD by DSM-5 criteria, with no medical treatment for ADHD and healthy controls aged between 18 and 60 years were included in the study. Written informed consent was obtained from each participant. Each participant was scheduled for a detailed psychiatric interview and administration of the scales. All scales and psychiatric interviews were conducted by a psychiatrist. All participants were of Turkish origin.

### 2.2. Instruments

Patients were evaluated using the Wender Utah Rating Scale (WURS) and Adult ADD/ ADHD DSM-IV-Based Diagnostic Screening and Rating Scale.

#### 2.2.1. Wender–Utah rating scale

This scale can be used to assess adults for ADHD with a subset of 25 questions associated with that diagnosis. The scale’s validity and reliability for Turkish individuals were determined by Oncü et al^[14]^ with a cutoff score of 36.

#### 2.2.2. Adult ADD/ADHD DSM-IV-based diagnostic screening and rating scale

This scale is a self-assessment scale and patients can complete the questionnaire after being appropriately informed. When developing the adult ADD/ADHD Scale, 18 symptoms of the diagnostic criteria in DSM-IV were reframed so patients could understand them. The first part of this scale had 9 inattention questions and the second part had 9 hyperactivity/impulsivity questions. The adult ADD/ ADHD Scale was found to be reliable and valid in the Turkish adult population by Gunay et al.^[15]^

Patients who scored 36 points or more on the WURS and gave an answer of 2 or 3 points to at least 6 of the 9 questions in the first and/or second parts of the adult ADHD Diagnosis and Evaluation Scale were diagnosed with ADHD.

### 2.3. Diagnostic criteria of premature ejaculation

PE was diagnosed according to DSM-5 criteria during the clinical interview with the participants by a psychiatrist. The DSM-5 defined PE as: “consistently ejaculating within 1 minute or less of vaginal penetration” over “at least 6 months” and “experienced 75% to 100% of the time.” The condition results in “clinically significant distress, sexual frustration, dissatisfaction, or tension between partners.” Moreover, “this condition is not better accounted for by another nonsexual mental disorder, medication or illicit substance use, or medical condition.”^[16]^

### 2.4. Measures

Also, the 2D and 4D of participants were measured with a digital caliper (0.01 mm sensitivity). The length of the second (index) finger (2D) and fourth (ring) finger (4D) was measured from the base of the proximal phalanx (metacarpophalangeal joint) to the tip of the distal phalanx, making sure the fingers were straight, not bent or curved.

### 2.5. Statistical analysis

Statistical analysis was performed using SPSS 24.0 Software (Armonk, NY: IBM Corp.). Descriptive data were obtained by using descriptive statistical methods such as mean, standard deviation, ratio, minimum, and maximum. Quantitative data comparison between groups was made using a *t*-test for parameters with a normal distribution. Pearson Chi-square test was used to perform qualitative data comparison. A *P*-value < .05 was considered statistically significant for all statistical tests.

## 3. Results

Of the 43 patients, 7 refused to participate, and 12 were excluded due to additional psychiatric and urological diagnoses. Twenty-four adult male ADHD patients who met the conditions of participation and 38 healthy controls were included in the study. The mean age of the patients was 28.67 ± 8.55 and the mean age of the healthy control group was 31.97 ± 8.11 (*P* > .05). Of the 24 patients with ADHD, 16 (66.7%) were combined subtypes, and 8 (33.3%) were inattentive subtypes.

The prevalence of PE in the ADHD group was 11 (45.8%), of which 8 (72.7%) were combined and 3 (27.3%) were inattentive subtype, while PE was detected in 2 (5.3%) of the control group.

The average age of ADHD patients with PE was 30.18 ± 8.81 and the average age of those without PE was 27.38 ± 8.45 (*P* > .05).

There was a statistically significant difference between the ADHD group and the control group in terms of right 2D length (*P* = .038) and 2D:4D (*P* = .004) (Tables 1 and 2). When those with PE were compared with those without PE, there was no statistically significant difference between the groups in terms of 2D lengths (*P* > .05) and 4D lengths (*P* > .05) (Table 2). Although there was no statistically significant difference (*P* > .05), both the right and left-hand 2D:4D are lower in ADHD with PE than in ADHD without PE (Table 3).

**Table 1:** The length of 2D and 4D in the ADHD group, in the control group, and regarding the presence of PE in adult ADHD.

	n	Right 2D			Right 4D			Left 2D			Left 4D		
		Mean	SD	*P*-value	Mean	SD	*P*-value	Mean	SD	*P*-value	Mean	SD	*P*-value
ADHD group	24	72.328	3.599	**.038** *	73.091	4.240	.654	71.908	3.258	.325	72.948	4.050	.589
Control group	38	69.968	5.091		72.533	5.463		70.841	5.212		72.253	6.022	
ADHD with PE	11	71.952	3.482	.645	72.736	3.764	.709	71.075	3.336	.261	72.482	3.328	.605
ADHD without PE	13	72.647	3.805		73.392	4.738		72.614	3.147		73.343	4.673	

Bold value indicates statistical significance.

2D = second digit, 4D = fourth digit, ADHD = attention deficit hyperactivity disorder, Mean = mean digit length in millimeters, PE = premature ejaculation, SD = standard deviation.

* *P* < .05.

**Table 2:** 2D:4D in adult ADHD and control group.

	n	Right 2D:4D			Left 2D:4D		
		Mean	SD	*P*-value	Mean	SD	*P*-value
ADHD group	24	0.9904	.0332	**.004** *	0.9868	0.0335	.534
Control group	38	0.9656	.0268		0.9812	.0354	

Bold value indicates statistical significance.

2D = second digit, 2D:4D = second to fourth digit ratio, 4D = fourth digit, ADHD = attention deficit hyperactivity disorder, Mean = mean digit length in millimeters, PE = premature ejaculation, SD = standard deviation.

* *P* < .05.

**Table 3:** 2D:4D regarding the presence of PE diagnosis in adult ADHD.

	n	Right 2D:4D			Left 2D:4D		
		Mean	SD	*P*-value	Mean	SD	*P*-value
ADHD with PE	11	0.9896	0.0374	.917	0.9816	.0413	.512
ADHD without PE	13	0.9911	0308		0.9912	.0262	

2D *=* second digit, 2D:4D *=* second to fourth digit ratio, 4D *=* fourth digit, ADHD *=* attention deficit hyperactivity disorder, Mean *=* mean digit length in millimeters, PE *=* Premature ejaculation, SD *=* standard deviation.

## 4. Discussion

In 1 of 2 studies that investigated the relationship between adult ADHD and PE, a total of 38 patients with PE were included in the study. The prevalence of ADHD has been reported at 42.1% in patients with lifelong PE.^[6]^ In the other study, ADHD was reported in 45.8% of 48 patients diagnosed with PE.^[10]^ In both studies, the study group consisted of PE patients, and the frequency of ADHD in PE was examined. Considering that the frequency of adult ADHD is 4%, the prevalence of adult ADHD with PE was reported to be approximately 10 times higher in the general population.

In this study, the study group is adult ADHD patients, and it is the first study to examine the frequency of PE in adult ADHD. PE has a prevalence of approximately 20% to 30% worldwide.^[5]^ In the present study, the prevalence of PE with adult ADHD (45,8%) was determined to be more than the general prevalence and the control group. PE was detected in 5.2% of the control group, which was similar to the general population. It also supports the above literature that reported the relationship between adult ADHD and PE.^[6,7]^

In this study, the mean age was 28.7 in the group. In a study conducted in Turkey, the prevalence of PE was found to be 9.2% in 1230 healthy men with a mean age of 27.3%.^[17]^ Considering the mean age, the prevalence of PE in adult ADHD was thought to be much higher (9.2% vs. 45.8%).

Corona et al associated delayed ejaculation with low male hormone levels. They reported that testosterone has a facilitating role in controlling the ejaculation reflex, independent of central or peripheral problems.^[18]^ Also, higher serum testosterone levels can shorten the duration of an erection. It has been reported that patients with PE have higher free testosterone and follicle-stimulating hormone levels compared to normal men, which supports the facilitating effect of testosterone in the control of the ejaculation reflex.^[8]^ In this study, the very high frequency of PE in adult ADHD suggested that it may be associated with high testosterone exposure in ADHD.

Both prenatal testosterone exposure and postnatal testosterone levels have been associated with the developing behavioral systems and neural circuitry. From childhood to adolescence, postnatal testosterone activation potentially affects neurodevelopment as well as behavioral and physiological change.^[19]^ There are various studies investigating the relationship between postnatal serum testosterone levels and ADHD. For example, Herguner et al^[20]^ reported that women with polycystic ovary syndrome, characterized by hyperandrogenism, showed more ADHD symptoms than control subjects. In addition, higher testosterone levels in plasma or saliva have been observed in children with hyperactivity and aggressive tendencies.^[21]^ Postnatal high testosterone levels in ADHD were thought to explain the fact that the frequency of PE in this study was much higher than in the normal population.

A lower 2D:4D generally indicates higher intrauterine testosterone exposure than estrogen.^[22]^ In several studies, more masculinized finger length rates (i.e., increased prenatal testosterone exposure) have been reported to be associated with ADHD symptoms.^[11,12]^ It is also supported by these studies that prenatal testosterone exposure contributes to the development of ADHD in children.

In 1 study, the role of prenatal and postnatal testosterone levels in ADHD was investigated. The study reported that higher prenatal testosterone exposure is associated with an increased risk of developing disruptive behavior disorders and that testosterone may exert different neurocognitive effects among girls and boys with ADHD.^[22]^ In this study, although there is no statistically significant difference, both left and right-hand 2D:4D were lower in ADHD with PE than in ADHD without PE. It can be thought that prenatal and postnatal high testosterone exposure in ADHD increases the incidence of PE with ADHD, in which postnatal testosterone exposure plays a role in its etiology.

It is thought that PE may also be caused by psychological stress factors such as anxiety, depressive disorders, and relationship problems. Psychopharmacological studies have suggested that PE is not caused by psychiatric disorders; on the contrary, lifelong PE is a neurobiological phenomenon and neurotransmitters such as dopamine and oxytocin may play a role in etiology in addition to the serotonergic system. However, it is reported that ADHD also has a complex pathogenesis and that abnormalities in the dopamine, noradrenaline, and serotonin systems may play a role in etiology.^[7,23]^ It has also been reported that the dopamine receptor D4 gene polymorphism plays a role in both ADHD and PE.^[23]^

Limitations of the study include a small study population, diagnosis of PE only according to DSM-5 criteria, and lack of serum testosterone levels. Also, sexuality is still a taboo subject in Turkey and the diagnosis of PE is made with subjective information, which can be considered another limitation. The inability to exclude the influence of potential confounders (e.g., age, comorbidities) and associations between variables is another limitation of the study.

## 5. Conclusions

In adults with ADHD, PE was found to be both more frequent and, although not statistically significant, lower the 2D:4D, suggesting that it might be possible that prenatal testosterone exposure and/or high testosterone levels may play a role in the etiology of both adult ADHD and PE. For this reason, it was thought that the frequency of coexistence is high because of the possible common etiology of these 2 clinical diagnoses.

In the English literature, there were only a few studies that investigated the frequency of ADHD in patients with PE. However, this study is the first research that investigated the frequency of PE in patients with adult ADHD. It is also the first study to investigate the relationship between this frequency and the 2D:4D, which is considered an indicator of fetal testosterone exposure.

In future studies, more advanced methods, such as regression analysis or multivariate analysis, can be used to control for potential confounders and to explore the relationships between variables more thoroughly. There is a need for studies in which the sample size is increased and hormone levels, such as serum testosterone and follicle-stimulating hormone, are also analyzed. It is also recommended to investigate the correlation between serum testosterone levels and the 2D:4D.

## Author contributions

**Conceptualization:** Gonca Ayse Unal, Selin Balki Tekin, Ayse Nur İnci Kenar.

**Data curation:** Gonca Ayse Unal, Ayse Nur İnci Kenar, Sezai Ustun Aydin, Alper Ziblak.

**Formal analysis:** Sezai Ustun Aydin.

**Investigation:** Gonca Ayse Unal, Alper Ziblak.

**Methodology:** Gonca Ayse Unal, Selin Balki Tekin, Sezai Ustun Aydin.

**Project administration:** Selin Balki Tekin, Ayse Nur İnci Kenar.

**Resources:** Gonca Ayse Unal, Selin Balki Tekin.

**Writing – original draft:** Gonca Ayse Unal, Selin Balki Tekin, Ayse Nur İnci Kenar, Sezai Ustun Aydin, Alper Ziblak.

**Writing – review & editing:** Gonca Ayse Unal, Selin Balki Tekin, Ayse Nur İnci Kenar, Sezai Ustun Aydin, Alper Ziblak.
